# Achromatic diffractive liquid-crystal optics for virtual reality displays

**DOI:** 10.1038/s41377-023-01254-8

**Published:** 2023-09-15

**Authors:** Zhenyi Luo, Yannanqi Li, John Semmen, Yi Rao, Shin-Tson Wu

**Affiliations:** 1 College of Optics and Photonics, University of Central Florida, Orlando, FL 32816 USA; 2 Goertek Electronics, 5451 Great America Parkway, Suite 301, Santa Clara, CA 95054 USA

**Keywords:** Displays, Imaging and sensing

## Abstract

Diffractive liquid-crystal optics is a promising optical element for virtual reality (VR) and mixed reality as it provides an ultrathin formfactor and lightweight for human factors and ergonomics. However, its severe chromatic aberrations impose a big challenge for full-color display applications. In this study, we demonstrate an achromatic diffractive liquid-crystal device to overcome this longstanding chromatic aberration issue. The proposed device consists of three stacked diffractive liquid crystal optical elements with specifically designed spectral response and polarization selectivity. The concept is validated by both simulations and experiments. Our experimental results show a significant improvement in imaging performance with two types of light engines: a laser projector and an organic light-emitting diode display panel. In addition, our simulation results indicate that the lateral color shift is reduced by ~100 times in comparison with conventional broadband diffractive liquid-crystal lens. Potential applications for VR-enabled metaverse, spatial computing, and digital twins that have found widespread applications in smart tourism, smart education, smart healthcare, smart manufacturing, and smart construction are foreseeable.

## Abstract (web summary)

An achromatic diffractive liquid crystal optics system has been demonstrated to overcome the longstanding chromatic aberration issue, which provides more compact optical components for a wide range of applications.

## Graphical abstract

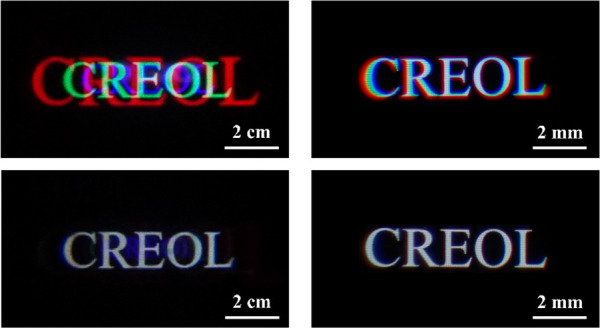

## Introduction

As a promising candidate for next-generation mobile platform, mixed reality (MR) such as Apple Vision Pro and Meta Quest Pro (both are passthrough virtual reality (VR) headsets) has the potential to revolutionize the ways we perceive and interact with various digital information. By providing more direct interactions with digital information, MR is one of the key enablers for metaverse, spatial computing, and digital twins that have found widespread applications in smart tourism, smart healthcare, smart manufacturing, and smart construction, just to name a few^1–5^. To further enhance the human factors and ergonomics of these near-eye displays, it is essential to improve the overall user experience, particularly for long-term wearing comfort. To achieve this goal, ultracompact formfactor and lightweight are urgently needed.

**Fig. 1:**
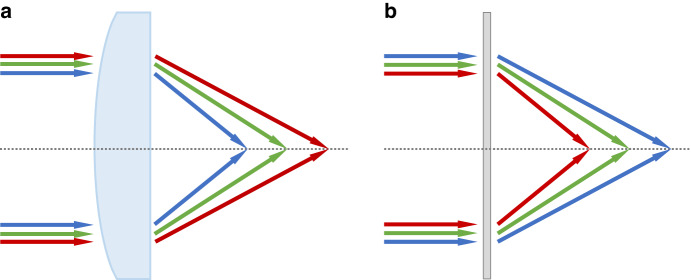
Chromatic aberrations of different lenses. **a** A refractive lens and **b** a diffractive lens for the RGB wavelengths

Refractive optics, such as a converging lens, produce phase difference based on the optical path difference. Despite the bulky formfactor, refractive optics are still widely used as optical combiners and imaging optics in recent near-eye display devices^6–8^. On the other hand, diffractive optics can provide the same phase pattern with a much thinner formfactor, but its chromatic dispersion is generally more apparent than that of refractive optics. Another distinct difference between refractive and diffractive optics is that their chromatic aberration behaviors are opposite^8^. Let us take the lens as an example. For a refractive lens shown in Fig. 1a, the focal length is dependent on the refractive index of the lens medium. According to Lensmaker’s formula, among the incident red, green, and blue (RGB) lights, the red color has the smallest refractive index so that its focal length is the longest, but the order is reversed for the diffractive lens as Fig. 1b depicts. The reason is that for a diffractive lens, the diffraction angle is proportional to the wavelength. That means, the red wavelength will have a larger angle, i.e., shorter focal length than the green and blue. An elegant approach to mitigate the chromatic aberration is to combine a diffractive optics with a refractive optics^8^. However, the chromatic aberration to be corrected in such a system originates from the refractive optics, while diffractive optics is used as a compensator. Thus, the whole system is still bulky and heavy. Metasurfaces offer a great potential solution as an achromatic diffractive optics, but currently the aperture size is still relatively small^9–11^. Moreover, the imaging quality and complex fabrication process remain to be overcome. Therefore, achieving a high-quality achromatic imaging performance while maintaining a compact formfactor and large aperture remains a big technical challenge. Practical applications of diffractive optics will be wide open if the above-mentioned issues can be solved.

Liquid crystal (LC) displays are ubiquitous nowadays, covering from microdisplay light engines for VR to large-screen TVs^12,13^. Besides displays, LCs also exhibit great potential in diffractive optics. Among various types of diffractive optics, LC-based Pancharatnam-Berry optical elements (PBOEs, also known as geometric phase optical elements) stand out because of their large aperture, lightweight, and simple fabrication process^14–19^. Unlike refractive optics, which uses optical path difference to produce phase patterns, LC-based PBOEs are patterned half-wave plates (HWPs), producing desired phase patterns by satisfying the half-wave condition along the thickness direction, which is typically several microns for a visible light. These LC-based PBOEs offer several advantages, such as high diffraction efficiency (nearly 100%), easy fabrication, polarization selectivity, and dynamic switching, making them a promising candidate for near-eye display applications. However, the diffraction angle of PBOEs depends on the wavelength, which in turn leads to a severe chromatic aberration.

A LC-based PBOE can be regarded as a patterned half-wave plate, and the underlying principle can be described using Jones matrix representation^20,21^. In the experiment, we use Mach-Zehnder Interferometer (MZI) to fabricate the PBOEs. The interference pattern of the two-beam MZI with opposite circular polarization becomes linearly polarized with the spatial distribution shown below:

1 $${{\exp }}\left(i\alpha \right)\left[\begin{array}{c}1\\ -i\end{array}\right]+{{\exp }}\left(i\beta \right)\left[\begin{array}{c}1\\ i\end{array}\right]=2{{\exp }}\left(i\frac{\alpha +\beta }{2}\right)\left[\begin{array}{c}{{\cos }}\left(\frac{\alpha -\beta }{2}\right)\\ {{\sin }}\left(\frac{\alpha -\beta }{2}\right)\end{array}\right]$$

where α and β is the phase profile of the two circularly polarized lights in the employed MZI, respectively. The phase pattern can represent a deflector, a lens or other profiles depending on the system design. Here, we introduce θ = (α − β)/2 as the azimuthal angle, which can be recorded by placing a photoalignment (PA) material that is sensitive to the polarization states at the interference plane for exposure. Afterwards, a birefringent LC monomer mixture is spin-coated on top of the PA film to create a uniform LC layer. The LC molecules align themselves along the azimuthal angle direction and the LC layer can be regarded as a phase retarder with a retardation ϕ = 2π*d*Δn/λ, where *d* is the LC layer thickness, Δn is the birefringence, and λ is the wavelength. When a circularly polarized light impinges the LC phase retarder, the polarization state of the outgoing light can be calculated using the Jones matrix as follows:

2 $$\frac{1}{\sqrt{2}}\left[\begin{array}{cc}{{\cos }}\theta & {{\sin }}\theta \\ -{{\sin }}\theta & {{\cos }}\theta \end{array}\right]\left[\begin{array}{cc}{e}^{-i\phi /2} & 0\\ 0 & {e}^{i\phi /2}\end{array}\right]\left[\begin{array}{cc}{{\cos }}\theta & -{{\sin }}\theta \\ {{\sin }}\theta & {{\cos }}\theta \end{array}\right]\times \left[\begin{array}{l}1\\ i\end{array}\right]=\frac{\sqrt{2}}{4}\times {e}^{-i\phi /2}\times \left(\left(1+{e}^{i\phi }\right)\left[\begin{array}{c}1\\ i\end{array}\right]+{e}^{-2i\theta }\left(1-{e}^{i\phi }\right)\left[\begin{array}{c}1\\ -i\end{array}\right]\right)$$

Here, we focus on the two circularly polarized terms at the right side of Eq. (2). The first term that remains the same handedness as the incident beam is the zeroth-order transmission and its intensity η_0_ equals to cos^2^(ϕ/2). The second term with an opposite handedness is the first-order transmission and its intensity η_1_ equals to sin^2^(ϕ/2)^18,22^. Since ϕ is jointly determined by *d*, Δn and λ, we can design PBOEs with desired spectra by choosing these parameters properly.

Equation (2) can be further simplified when the LC layer thickness *d* satisfies the half-wave condition (dΔn = λ/2) for a desired wavelength. Under such condition, ϕ = π, and Eq. (2) is reduced to:

3 $$\frac{1}{\sqrt{2}}\left[\begin{array}{cc}{{\cos }}2\theta & {{\sin }}2\theta \\ {{\sin }}2\theta & -{{\cos }}2\theta \end{array}\right]\left[\begin{array}{c}1\\ \pm i\end{array}\right]=\frac{1}{\sqrt{2}}\left[\begin{array}{c}1\\ \mp i\end{array}\right]{e}^{\pm 2i\theta }$$

Only the term that represents the first-order transmission exists in the simplified Eq. (3). When a circularly polarized light passing through the LC-based PBOE, not only the polarization state is switched, but the output beams also acquire an additional positive or negative 2θ phase, and the recorded phase pattern can be reconstructed.

**Fig. 2:**
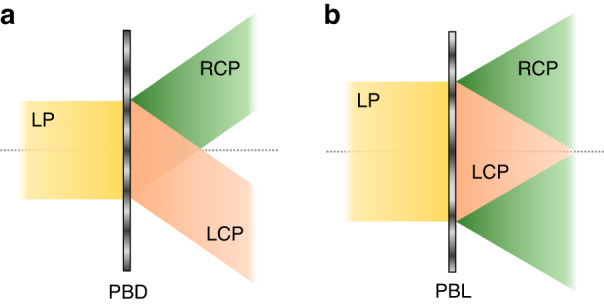
Polarization-dependent response of PBOEs. **a** A PBD and **b** a PBL. LP linear polarization, RCP righthanded circular polarization, LCP lefthanded circular polarization

Figure 2 illustrates the polarization-dependent behaviors of the commonly used LC-based PBOEs: Pancharatnam-Berry deflector (PBD) and Pancharatnam-Berry lens (PBL). When a linearly polarized (LP) light impinges the PBD, RCP and LCP will be deflected to an opposite angle, as Fig. 2a depicts. Meanwhile, the output beam after PBL can be diverged or converged depending on the polarization state shown in Fig. 2b. The optical response of the two circular polarizations can be reversed by simply flipping the PBL.

In this paper, we propose an achromatic diffractive LC optical element by stacking three PBOE films together. Specifically, we design the spectra of LC-based PBOEs appropriately to control the polarization states of the incident RGB lights. Taking the advantage of polarization selectivity, the LC-based PBOEs offer a desired negative or positive phase compensation to different colors, which in turn corrects the chromatic aberrations. Both simulation and proof-of-concept experiment are conducted to verify the effectiveness of our approach. Our simulation results indicate that the proposed achromatic LC lens system reduces the lateral color shift by about 100 times, compared to a broadband diffractive LC lens at 50° field angle, which corresponds to 100° field of view (FOV) for a VR headset. Our new method overcomes the longstanding chromatic aberration problem of diffractive optics, which will accelerate the practical applications of these diffractive elements for advanced display systems.

## Results

**Fig. 3:**
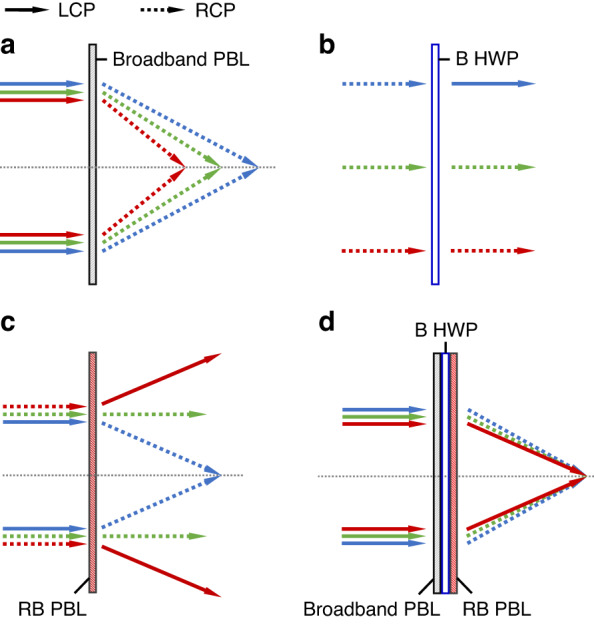
Working principles of our proposed achromatic LC diffractive lens. Optical response of RGB lights to **a** broadband PBL, **b** B HWP, **c** RB PBL, and **d** our proposed achromatic LC diffractive lens system. Here, solid lines represent LCP and dashed lines represent RCP

Based on the PBOE properties discussed above, we develop an achromatic diffractive LC optics system consisting of three components shown in Fig. 3. The system can be applied to both deflectors and lenses. Here, we use an achromatic diffractive LC lens system as an example to elucidate the working principles. For simplicity but without losing generality, let us assume the input is an LCP light. As Fig. 3a illustrates, the LCP lights (solid lines) first pass through a broadband PBL, which is effective to all the RGB lights. Such a broadband PBOE can be obtained using multi-twist structures^23,24^. This broadband PBL provides different optical powers to the input RGB beams. As a result, a severe chromatic aberration occurs, as Fig. 3a shows. The red light experiences the highest optical power while the blue light has the lowest one as discussed before. Besides, the polarization states of RGB lights are switched to RCP (dashed lines) after passing through the broadband PBL.

The second component is a B HWP with a uniform phase profile; its functionality is shown in Fig. 3b. The B HWP is placed after the broadband PBL. Such a B HWP is designed to only convert the polarization state of the blue light from RCP to LCP but causing no effect to the red and green lights. Theoretically, such a HWP can also be designed to be effective only to the red light. However, according to our simulation results, such an optimized R HWP not only exhibits nearly 100% efficiency at the designed wavelength λ = 639 nm, but also shows about 20% efficiency at λ = 457 nm, which could result in severe ghost images due to the polarization impurity. In contrast, the optimized B HWP for 457 nm shows less than 5% polarization-conversion efficiency and a broader low-efficiency band in the vicinity of 639 nm, which is preferred because the VR light engine (LCD or OLED) could have a 30-nm emission bandwidth.

The third component is RB PBL, which is effective only for the red and blue lights. With the B HWP controlling the polarization state, the handedness of red light (RCP) and blue light (LCP) is opposite before they reach the RB PBL. Due to polarization selectivity, the RB PBL diverges the red light but converges the blue light, as Fig. 3c shows. By stacking these three PBOEs together as shown in Fig. 3d, our achromatic LC diffractive optics system is achieved. The thickness of each PBOE is only a few microns, which ensures a compact formfactor for the entire system.

**Fig. 4:**
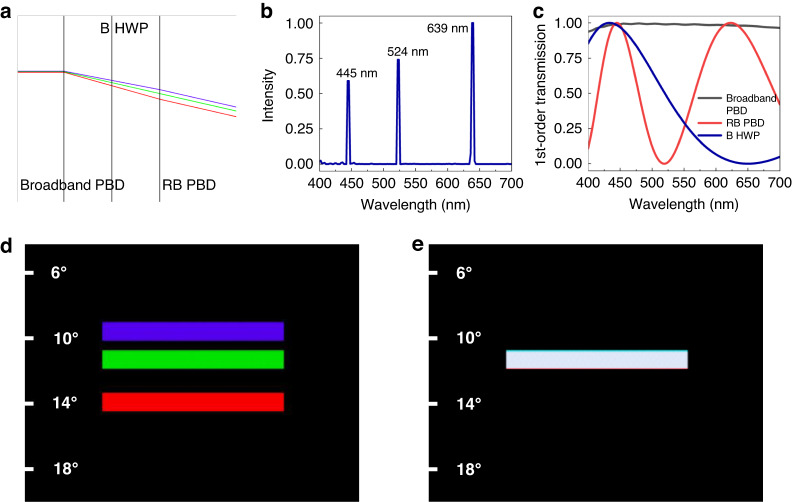
Simulations for the achromatic LC deflector system. **a** Optical layouts, **b** emission spectrum of the source, **c** simulated 1^st^-order efficiency of the broadband PBD, RB PBD, and B HWP, and simulated results of **d** the broadband PBD and **e** our proposed achromatic system consisting of 25% blue, 32% green, and 43% red colors, as an example

The achromatic performance of our proposed system is jointly governed by the numerical relationship between the broadband and RB components and their spectra. Therefore, the design can be applied to both deflector and lens equally well. For simplicity, here we use a LC-based PBD as an example to prove the concept of an achromatic LC deflector system. Figure 4a depicts the optical layout of the system and the simulated results. The incident RGB lights are diffracted to different angles after passing through the broadband PBD. The diffraction angle (*θ*) depends on the wavelength (*λ*) as follows:

4 $$\frac{{{{\sin }}\theta }_{R}}{{\lambda }_{R}}=\frac{{{{\sin }}\theta }_{G}}{{\lambda }_{G}}=\frac{{{{\sin }}\theta }_{B}}{{\lambda }_{B}}$$

Next, a B HWP is introduced to convert the polarization state of blue light. As a result, before reaching the RB PBD, the red light and blue light exhibit an opposite handedness. Subsequently, the diffraction angle after the RB PBD becomes negative for red light and positive for blue light. The different diffraction angles can be compensated by a proper design of the two PBDs. The emission spectrum of the light source utilized in our simulation is presented in Fig. 4b, with R = 639 nm, G = 524 nm, and B = 445 nm, respectively. The simulated diffraction angle of each component for the normal incidence is listed in Table 1.

**Table 1:** Simulated diffraction angles of each PBOE at the specified RGB wavelengths

	639 nm	524 nm	445 nm
Broadband PBD	14.1°	11.5°	9.7°
B HWP	0°	0°	0°
RB PBD	−2.5°	0°	1.7°
Achromatic system	11.5°	11.5°	11.5°

In addition to diffraction angle, the spectra also play a crucial role for the achromatic performance. The 1^st^-order transmission spectra of the broadband PBD, B HWP and RB PBD are plotted in Fig. 4c. Different from conventional PBOEs with oscillating transmission spectra on the wavelength, the broadband PBD has a multi-twist structure and shows a high efficiency in the visible spectral range. The thickness of RB PBD is 8.7 μm when using a LC reactive mesogen RM257 with Δn = 0.179. The spectrum of B HWP, with a thickness of 3.6 μm, is represented by the solid blue line. From Fig. 4c, the polarization state of a portion of green light will also be flipped by the B HWP. Fortunately, the RB PBD only deflects blue and red lights, so that the polarization states of green light will not cause further chromatic aberrations. The simulation results of broadband PBD are illustrated in Fig. 4d. As expected, RGB lights are deflected to different angles, resulting in a severe chromatic aberration. After employing the B HWP and RB PBD, the deflection angles in Fig. 4e are the same for all the incoming RGB lights, demonstrating the effectiveness of our design. The simulated optical efficiency for RGB lights is 62.4%, 59.8%, and 53.1%, respectively. Such an optical loss mainly originates from the incomplete polarization conversion of the broadband PBD. As discussed before, conventional PBOEs with a single LC layer can convert the circular polarization of a monochromatic incident light to an opposite handedness with an efficiency of nearly 100%. However, to achieve broadband spectrum using a multi-twist structure, the polarization conversion is incomplete, i.e., LCP and RCP coexist in the output beam. To eliminate the ghost image caused by the incomplete polarization conversion of the broadband PBD, we can employ two circular polarizers (CPs) to absorb the unwanted handedness, as will be discussed later in the experiment section.

In addition to simulations, we experimentally demonstrated an achromatic LC diffractive lens system. Similar to the deflector system, the lens system consists of three components, a broadband PBL, a B HWP and an RB PBL. The focal lengths of these two PBLs should be properly chosen by considering the input wavelengths of blue light and red light to eliminate the chromatic aberration problem perfectly. To obtain a desired phase pattern, we used MZI to record the interference patterns on a photoalignment layer that is sensitive to the polarization states of the incident beams as we discussed before.

**Fig. 5:**
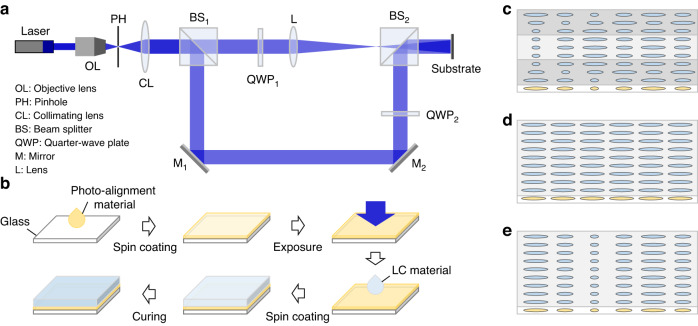
Fabrication process. **a** MZI for PBL fabrication. **b** Fabrication flowchart of PBOEs. LC layer structure of **c** broadband PB lens, **d** B HWP, and **e** RB PBL

Figure 5a depicts the optical setup for the exposure process. The collimated laser beam (λ = 457 nm) was first split into two beams using a beam splitter (BS). Each beam was then converted to an opposite circular polarization using two quarter-wave plates (QWPs). One beam carrying lens phase pattern was recombined with another collimated beam for interference at the sample by the second BS. The distance between the focused point and substrate along the optical axis is the exposure focal length. In most cases, the exposure wavelength is different from the operating wavelengths since the absorption peak of our photoalignment material is located at blue or UV light range. However, the fabricated PBOEs can be effective for green or red light depending on the design. The approximate relationship between optical power K and wavelength λ for PBLs is shown below:

5 $$\frac{{K}_{{expose}}}{{\lambda }_{{expose}}}=\frac{{K}_{R}}{{\lambda }_{R}}=\frac{{K}_{G}}{{\lambda }_{G}}=\frac{{K}_{B}}{{\lambda }_{B}}$$

The fabrication process of PBOEs is illustrated in Fig. 5b. Firstly, the photoalignment material was dissolved and spin-coated onto a clean glass substrate. Brilliant Yellow (BY; an organic diazo dye) and SD1 (sulfonic dye 1) are two commonly used photoalignment materials^25,26^. Both BY and SD1 offer excellent photoalignment quality. However, BY, as its name indicates, shows a slightly yellowish color, which may affect the clear state of the displayed images. On the other hand, SD1 exhibits better stability for practical applications. In our concept-proof experiment, we chose BY simply because it is commercially available, and it offers an excellent photoalignment quality. Subsequently, the photoalignment layer was exposed in the MZI using a 457-nm laser. The exposure focal length of the broadband PBL and RB PBL is 5 cm and 28 cm, respectively. The B HWP, which is designed to switch the polarization state without introducing any phase modification, was exposed to a collimated linearly polarized light, which exhibited a uniform phase profile. Reactive mesogen RM257 was utilized to create uniform LC layers after spin coating. Finally, the PBOEs were exposed to UV light for stabilization. After polymerization, their thermal properties, such as temperature dependent birefringence, are much stable. Moreover, UV stability is another issue for liquid crystal devices. Fortunately, for VR applications, the UV components in the microdisplay light engines, such as LCDs and OLED-on-silicon are extremely weak. Besides, the LC layers are fabricated on glass substrates, which help block the harmful incident UV light, if any. Therefore, the effects of UV light on the LC lenses should be negligible.

The transmission spectra of these PBOEs determine the operating range of the elements, thus, they are also crucial to the achromatic system. Specifically, the first PBL should be broadband and effective to all the input RGB lights. To achieve these goals, we used a multi-twist structure, and the total LC layer thickness is about 3.1 μm^23^. The LC layer structure of broadband PBL is illustrated in Fig. 5c. The adoption of chiral dopant in the LC mixture helps build a helical structure in the first and third LC layers. The spectra of B HWP and RB PBL are governed by the principle described in Eqs. (2) and (3), where the LC layer thickness and the LC birefringence jointly determine the spectra. Our calculation shows that using RM257 with Δn = 0.179, the optimal thickness of the B HWP should be about 6.7 μm to switch the polarization state of blue light, while preserving the handedness of the red light. Similarly, the optimal thickness of the RB PBL is around 8.7 μm, which makes it effective for blue and red but not for green light. The LC layer structures of B HWP and RB PBL are illustrated in Fig. 5d, e. The LC directors follow the photoalignment patterns at the bottom substrate. The focal length of PBL for polarization selectivity at R = 639 nm, G = 524 nm, and B = 445 nm is included in Table 2. Because only two PBLs are used in the designed system to eliminate the power difference between red and blue lights, the optical power of green light can slightly deviate from the other two colors. This can be resolved by selecting an appropriate wavelength for green light that ensures identical powers for all the three colors.

**Table 2:** Focal lengths of designed PBLs for RGB light

	457 nm	639 nm	524 nm	445 nm
Broadband PBL	5 cm	3.58 cm	4.36 cm	5.13 cm
RB PBL	28 cm	−20.03 cm	Infinity	28.76 cm
Achromatic system		4.35 cm	4.36 cm	4.36 cm

**Fig. 6:**
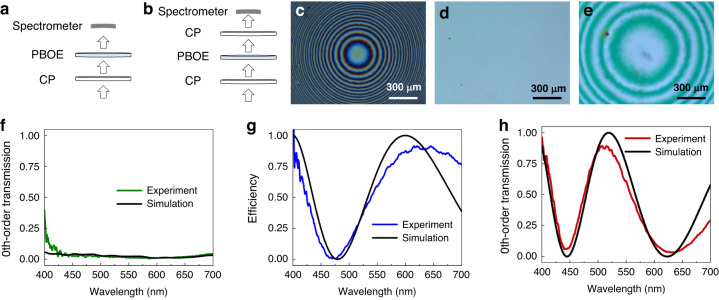
Characterizations of the fabricated PBOES. Setup for measuring 0^th^-order of **a** broadband PBL, RB PBL and **b** B HWP. Polarizing optical microscope images of **c** broadband PBL, **d** B HWP, and **e** RB PBL. Simulated and measured transmission spectra of **f** broadband PBL, **g** B HWP, and **h** RB PBL

We conducted simulation and measured the transmission spectra of the PBOEs, and the results are shown in Fig. 6, together with their corresponding polarizing optical microscope images. The setup for measuring the 0^th^-order transmission of PBOEs is sketched in Fig. 6a, b, respectively. The emitted light was converted to circular polarization by a CP before passing through the PBOE. For the LC lenses, the 1^st^-order diffracted light was converged or diverged and could not be collected by the spectrometer, so that we only measured the 0^th^-order transmission. The polarizing optical microscope images of these three PBOEs are shown in Fig. 6c–e. From Fig. 6f, the broadband PBL exhibits a minimal leakage throughout the visible region, suggesting most of the light is diffracted by the broadband PBL as expected. To measure the polarization conversion efficiency of B HWP, we added another CP (QWP + LP) with same handedness above the PBOE as Fig. 6b shows. The light passing through the PBOE was converted to linear polarization by the QWP and then transmitted through the linear polarizer. The 1^st^-order transmitted light was switched to an opposite handedness by the B HWP and was finally absorbed by the second CP with the same handedness as the first CP. Figure 6g depicts the measured polarization-conversion efficiency of B HWP alongside the simulated spectrum. Figure 6h displays the simulated and measured 0^th^-order transmission spectra of the RB PBL. The agreement is reasonably good. There are two valleys in the blue and red regions, indicating a high 1^st^-order diffraction efficiency in these two spectral bands. Most of the green light is not diffracted. Thus, the RB PBL is only effective for blue and red lights. Additionally, we find the polarization state of the green light at 524 nm is partially switched by the B HWP. However, the RB PBL is unable to provide any optical power for green light regardless of its polarization state as shown in the 0^th^-order transmission spectrum of RB PBL (Fig. 6h).

The stacked achromatic LC diffractive lens system consists of three planar elements in contact, and the total thickness of the entire system in our experiments is about 3 ~ 4 mm. However, most of the thickness is from the employed glass substrates (1-mm thickness) because all the three LC components were spin-coated onto these glass substrates. The effective thickness of each LC layer is only a few microns. Therefore, the formfactor of the achromatic system can be made much thinner by using a thinner substrate for each component. Nowadays, ultrathin (<100 μm) glass substrates are commercially available.

**Fig. 7:**
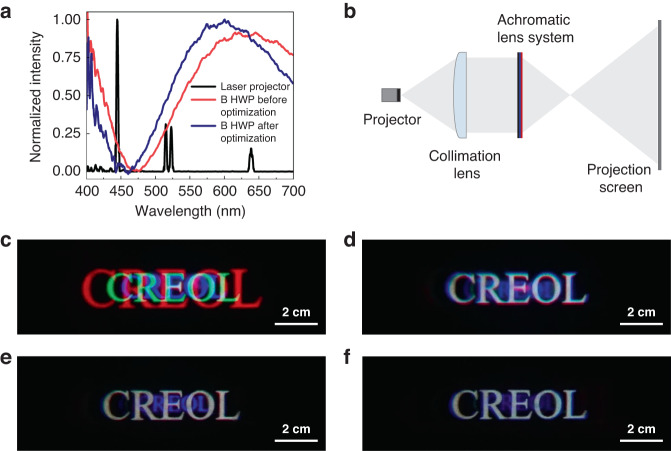
Experiments using a laser projector as the light engine. **a** Spectra of the employed laser projector and the B HWP, **b** optical setup for the imaging process with a laser projector, **c** captured image using a broadband PBL, **d** captured image using our achromatic LC lens system, **e** captured image using our achromatic LC lens system with two circular polarizers to eliminate the ghost image, and **f** captured image after optimizing the transmission spectrum of B HWP

**Fig. 8:**
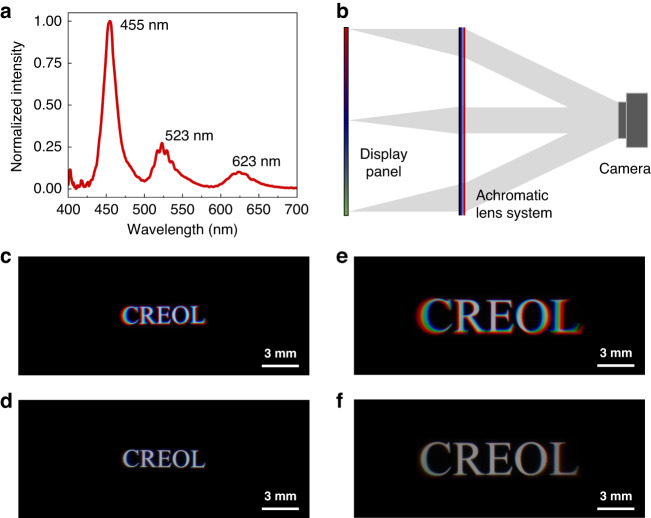
Experiments using an OLED display as the light engine. **a** Emission spectra of the employed OLED panel, **b** optical setup for the imaging process with an OLED panel, the captured image using a broadband PBL with **c** 23^o^ FOV and **e** 43^o^ FOV. Captured image using our achromatic LC lens system with **d** 23^o^ FOV and **f** 43^o^ FOV

After measuring the spectra and efficiency of the fabricated PBOEs, we evaluated the imaging performance of the achromatic LC diffractive lens system using two light sources: a laser projector (Sony MP-CL1A, Fig. 7) and an OLED display from a smartphone (Fig. 8). The obtained results are compared to those using a broadband PBL, which exhibits severe chromatic aberrations as expected from diffractive optics. Figure 7a shows the measured emission spectrum (black lines) of the laser projector. In the projection system shown in Fig. 7b, the output beam from the laser projector was first collimated before passing through the stacked achromatic LC lens system. The real images produced by this system were then projected onto a white surface, and the projected images were captured by a camera under a dark environment. Severe chromatic aberration resulting from the broadband PBL is clearly observed in Fig. 7c. The color performance fails to meet the requirements for practical applications.

Next, we evaluated the performance of our proposed achromatic LC lens system under the same configuration. As Fig. 7d shows, the chromatic aberrations are dramatically suppressed. However, the undesired red spots in the central region degrade the imaging performances of our achromatic system. These spots are zeroth-order ghost images from the high-intensity laser projector. Another cause for the ghost images is that the polarization conversion efficiency of the broadband PBL is not 100%. As discussed before, when a circularly polarized light passes through a conventional LC-based PBOE, the output beam not only acquires an additional phase, but the polarization state is also switched. In principle, the efficiency for the polarization switching can reach almost 100%. However, due to the adoption of multi-twist structure, the polarization-conversion efficiency of our broadband PBL cannot achieve 100%, which indicates a small portion of circularly polarized light with undesired handedness existed in the output beams after the broadband PBL and finally generated the ghost images. To eliminate the ghost images, we employed two circular polarizers (CPs) to absorb the unwanted light. Each CP consists of a linear polarizer (LP) and a QWP. We stacked the two CPs together with following order: QWP/LP/LP/QWP, i.e., the linear polarizer side is facing each other. For simplicity, these two CPs can be combined into one: QWP/LP/QWP.

The optical axes of two linear polarizers were aligned carefully to be parallel. Then, we placed this element after the broadband PBL to absorb the unwanted circular polarization. The improved imaging results are illustrated in Fig. 7e; the red ghost images were eliminated completely as expected. However, the blue ghost image still exists, resulting from mismatched spectrum between our B HWP and the laser projector. In our original design, the B HWP should have a high efficiency for the blue light but very low efficiency for the red light. The spectra of PBOEs are determined by the half-wave condition which depends on the LC birefringence and the LC layer thickness. The transmission spectrum of the B HWP used in Fig. 7e is shown as the red line in Fig. 7a. To measure the efficiency of such a B HWP, we placed the sample between two circular polarizers with the same handedness and aligned in the same direction. Ideally, the transmittance of the B HWP at the blue light from the projector (445 nm) should be 0%, indicating the polarization state is converted completely. But as Fig. 7a shows, there is still about 20% of the blue light leakage, which is responsible for the observed ghost images in Fig. 7e. We also notice that the efficiency of B HWP at λ = 639 nm is only about 85%, which means the polarization state is not pure and may also produce ghost images. The reason we do not see the red ghost images in Fig. 7e is threefold: 1) the ghost image size of red light is larger than that of the blue light, 2) it is attenuated more quickly, and 3) its intensity is much weaker than that of the blue light. To eliminate the blue ghost, we fabricated another B HWP to better match the spectrum with the laser projector at 445 nm, as the blue curve depicts in Fig. 7a. The captured image is shown in Fig. 7f, where the blue ghost image is suppressed substantially.

In addition to the laser projector, we also investigated the achromatic behavior with an OLED panel, whose emission FWHM (full width at half maximum) is wider, as Fig. 8a depicts. Active-matrix OLED has been used in Sony’s PlayStation and micro-OLED has been used in Apple’s Vision Pro. To prove concept, we used an OLED smartphone as the light engine, although it is not an ideal source for VR because its radiation cone is too wide. The OLED panel was placed at the focal point of the imaging lens so that the resulting images are virtual, as illustrated in Fig. 8b. In experiment, we first tested the imaging performance of a broadband PBL. As expected, a severe chromatic aberration occurs, as shown in Fig. 8c, e, where the corresponding FOV is 23^o^ and 43^o^, respectively. Such a strong chromatic aberration arises from the wavelength dependent optical power of the diffractive PBL. Because the emission spectrum of the OLED panel (Fig. 8a) is different from that of the laser projector, we fabricated another RB PBL with a focal length of 32 cm at λ = 457 nm to match the OLED’s blue spectrum. Next, we evaluated the performance of our proposed achromatic system with the new RB PBL. The captured images are shown in Fig. 8d, f. As expected, the chromatic aberration is dramatically suppressed. In Fig. 8f, we can still see a very dim color dispersion at the left and right edges. This is because the OLED smartphone is designed to have a wide viewing angle. If we could narrow down the emission cone of the OLED panel, the weak chromatic aberration can be eliminated.

Different from above-mentioned projector system, the ghost images are hardly observed in the OLED system. The main reason is the different imaging processes in these two systems. The projector system forms a real image on a white screen. The incident light is collimated, and the blue stray light can still be projected to the screen to form a clear real image because the projection distance can be quite flexible for the projection system. However, the images in the OLED system are virtual and the image planes of signal and ghost are in different positions. Especially, the OLED panel is placed near the focal point of the imaging lens so that the distance between the two image planes can be quite far. Considering the limited depth of focus of our camera, the blue ghost is not able to form a clear image on the detector. Therefore, we can hardly see ghost images in the OLED system.

Our approach works well for both laser projector and OLED light engines. It is worth mentioning that the field angle in the imaging space is not the same as the incident angle onto the achromatic lens system. As Fig. 8b shows, the incident angle in object space is much smaller than the angle in imaging space. For the emitted light from edge pixels of the display panel in a VR system, only the emission cone with a small polar angle can finally reach the eye pupil^27^. The FOV in our system is currently limited by the *f*/# of the fabricated PBL. The diameter of these PBOEs in our experiments is about 16 mm, corresponding to a *f*/# of 2.7.

**Fig. 9:**
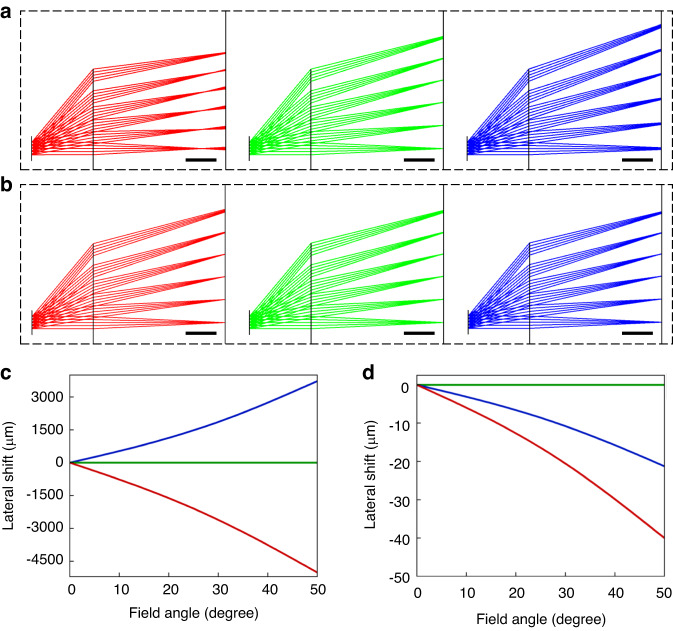
Simulated lateral color shift. Optical layout of **a** the broadband PBL system and **b** our proposed system. The scale bar is 1 cm. Lateral color shift of **c** the broadband PBL system and **d** our proposed system

From practical application viewpoint, wider FOV is highly desirable for near eye displays. Therefore, to access the achromatic performance of the proposed system under large FOVs, we conducted simulations using Zemax to analyze the lateral color shift. A large field angle of 50°, equivalent to a 100° FOV, was used in the simulation. Results are plotted in Fig. 9; here, we compare the performances of our proposed achromatic LC diffractive lens system with that of a broadband PBL.

Hologram surfaces in the sequential mode of Zemax were used to simulate the dispersion of diffractive optics. The construct wavelength and focal length can be easily defined as the same condition in our experiment. The broadband PBL was simulated using one hologram surface. Figure 9a shows the optical layout of the broadband PBL system where the RGB rays exhibit different paths, indicating the root cause of the chromatic aberrations in diffractive optics. The components in each layout are pupil, imaging optics, and display panel from left to right. The achromatic LC lens system was achieved by using two hologram surfaces with the same experimental construct condition. Figure 9b shows the optical layout of our proposed system where RGB rays follow almost identical paths, indicating the chromatic aberration has been suppressed significantly. Furthermore, we conducted a quantitative analysis of the color performance, as displayed in Fig. 9c, d. In these figures, the green light is used as a reference. In the case of broadband PBL (Fig. 9c), as the field angle increases, the lateral shift of blue and red beams gradually increases. At 50°, the lateral shift exceeds 4.5 mm for the red light and over 3 mm for the blue light. In contrast, in our proposed system the shift is less than 40 μm. The improvement is about 100 times at large field angles.

## Discussion

The *f*/# of our fabricated lens system is 2.7. The ability to produce PBLs with a smaller *f*/# has been demonstrated^28–30^. Therefore, the fabrication of PBLs with a larger aperture is feasible. The spectra of the B HWP, RB PBD, and PBL are determined by the half-wave conditions. Our lab only has RM257 whose birefringence is 0.179. If we can use a higher birefringence reactive mesogen, then the required LC layer thickness to achieve the half-wave condition will be reduced. A thinner LC layer not only requires fewer times of spin-coating processes but also leads to a better LC alignment. As a result, the PBOE efficiency is improved, which in turn suppresses the ghost images observed in our experiments. In addition to optimizing the reactive mesogen and the fabrication process, using a microdisplay with a narrower FWHM that is located inside the high-efficiency region of PBOEs also helps mitigate ghost images. Display devices with a smaller viewing cone ( ± 20^o^ vs. ±80^o^ for the OLED smartphone we used) for VR systems also improve the achromatic behavior of our proposed LC lens system because the efficiency of PBOEs decreases as the incident angle increases.

Image quality of the diffractive LC lenses is mainly determined by the phase pattern during the exposure process. Optimizing the phase pattern can improve the imaging performance of the LC lens system. In our experiment, Mach-Zehnder Interferometer with a single lens is used for the exposure process to demonstrate the achromatic behavior, which limits the design freedom of the phase pattern. Another phase patterning method, called direct writing, can provide the capability for the alignment of more complex phase patterns^31^. On the other hand, the imperfect polarization conversion caused by the off-axis incident light onto the LC optics can also degrade the image quality especially at the marginal area of the field. The polarization impurity at large field angles results in stray light, which in turn reduces the image contrast. Fortunately, the acuity of human eyes achieves the highest in the fovea region (~ ± 5°) and drops rapidly as the field angle increases^32^. Therefore, although the diffractive LC lenses cannot achieve an outstanding image quality at large incident angle to the pupil, they are still acceptable for the VR systems considering the small fovea region.

Our approach can be extended to other types of diffractive optics which do not exhibit polarization-selective response as PBOEs by keeping the same polarization states of the output RGB lights after the diffractive optics. This is easy to achieve from the emission side or adding a circular polarizer after the diffractive optics, and then using B HWP and RB PBL (or PBD) with a proper phase profile to correct the chromatic aberrations.

In present VR headsets, Fresnel lens and pancake lens are two major imaging optics. Stray light is a big issue for Fresnel lens because its surface has grooves. Moreover, the long object distance also leads to a larger formfactor. Therefore, pancake lens^6,18^ has been developed to reduce the formfactor and applied to recent MR headsets, such as Meta Quest Pro and Apple Vision Pro. To achieve good imaging quality within a large FOV, pancake lenses are designed to be cemented doublet or even triplet to correct the monochromatic aberrations. However, the cemented pancake lens also suffers from the disadvantages of refractive optics such as bulky formfactor and heavy weight. Fortunately, both pancake lens and our achromatic LC lens are polarization-dependent and can be combined to reduce the formfactor and the weight. Specifically, the achromatic LC lens can replace the refractive eye lens near the pupil. Meanwhile, our LC lenses have the potential to achieve more complex phase patterns to correct the aberrations with direct-writing system for the alignment.

In conclusion, we propose an achromatic LC diffractive optics system to overcome the longstanding chromatic aberration issue. The effectiveness of this concept is demonstrated through simulations for deflector system and experiments for lens system. The proposed system is composed of three PB optical elements with specifically designed spectral response, which is achieved by the precise control of LC layer thickness. Our experimental results indicate a significant improvement in imaging performance with different light engines, including a laser projector and an OLED panel. Our simulation results show that the lateral color shift is reduced by ~100 times compared to a conventional broadband PBL. Thanks to the use of polarization selective PB optical elements, our new approach enables an achromatic imaging performance while maintaining an ultrathin formfactor. This advancement is expected to open a new door for more compact optical components for beam steering, imaging, and display applications.

## Material and methods

### Materials

The photoalignment material used in our experiments is Brilliant Yellow (BY) from Tokyo Chemical Industry Co., Ltd. BY powders is dissolved in dimethyl-formamide with a weight concentration of 0.2%. The mixed solution is filtered using a 0.2-μm Teflon syringe before spin-coated onto the glass substrates. The LC mixture is composed of solvent toluene and precursor which contains LC monomer RM 257 purchased from Jiangsu Hecheng Advanced Materials Co., Ltd, surfactant Zonyl 8857 A from Dupont, and photo-initiator Irgcure 651 from BASF.

### Methods

The 1-mm-thick glass substrates were purchased from Fisher Scientific. The substrate was cleaned using ethanol and then treated by UV-Ozone for 5 minutes before the spin-coating of BY solutions. The humidity of the environment for spin-coating was controlled to be under 40%^33^. The BY layer on the glass substrate was exposed to a 457-nm laser (Cobolt Twist^TM^) with 200-mW output power for 2 minutes. We preheated the LC mixture on a hot plate stage at 70 °C before spin-coating as the viscosity decreases with increased temperature. To ensure the LC layer uniformity, the coating speed was kept above 2000 rpm in experiment and the dilution ratio of LC mixture was between 1:3 and 1:5. We also put the substrates with LC layers on top of the hot plate right after the spin-coating process for several seconds to obtain better alignment. Detailed recipes are summarized in Table 3.

**Table 3:** Materials and coating speed for PBOE fabrication

		Solute	Solvent	Solute: Solvent	Coating speed (rpm)
	PA layer	Brilliant yellow	Dimethyl-formamide	1:499	500 (5s) + 3000 (30s)
Broadband PBL	1^st^ layer	Zonyl 8857A (0.92%)	Tolune	1:4.8	2450 (30s)
		S811 (1.79%)			
		RM257 (95.19%)			
		Irgacure 651 (2.10%)			
	2^nd^ layer	Zonyl 8857A (0.96%)		1:3.9	800 (5s) + 3000 (30s)
		RM257 (96.86%)			
		Irgacure 651 (2.18%)			
	3^rd^ layer	Zonyl 8857A (0.95%)		1:3.8	700 (5s) + 3000 (30s)
		R811 (1.68%)			
		RM257 (95.98%)			
		Irgacure 651 (1.39%)			
B HWP	1^st^ and 2^nd^ layer	Zonyl 8857A (0.2%)		1:3	2000 (30s)
		RM257 (96.8%)			
		Irgacure 651 (3.0%)			
	3^rd^ to 5^th^ layer	Zonyl 8857A (0.2%)		1:4.3	
		RM257 (96.8%)			
		Irgacure 651 (3.0%)			
Optimized B HWP	1^st^ and 2^nd^ layer	Zonyl 8857A (0.2%)		1:3	
		RM257 (96.8%)			
		Irgacure 651 (3.0%)			
	3^rd^ and 4^th^ layer	Zonyl 8857A (0.2%)		1:3.5	
		RM257 (96.8%)			
		Irgacure 651 (3.0%)			
RB PBL	1^st^ to 5^th^ layer	Zonyl 8857A (0.2%)		1:3	
		RM257 (96.8%)			
		Irgacure 651 (3.0%)			
	6^th^ layer	Zonyl 8857A (0.2%)		1:4.3	
		RM257 (96.8%)			
		Irgacure 651 (3.0%)			

## Data Availability

All data needed to evaluate the conclusions in the paper are present in the paper. Additional data related to this paper may be requested from the authors.
